# Association of skeletal muscle mass, kidney disease and mortality in older men and women: the cardiovascular health study

**DOI:** 10.18632/aging.202135

**Published:** 2020-11-02

**Authors:** Nicholas T. Kruse, Petra Buzkova, Joshua I. Barzilay, Rodrigo J. Valderrabano, John A. Robbins, Howard A. Fink, Diana I. Jalal

**Affiliations:** 1 Division of Nephrology, Department of Internal Medicine, Carver College of Medicine, Iowa City, IA 52242, USA; 2 Department of Biostatistics, University of Washington, Seattle, WA 98195, USA; 3 Division of Endocrinology, Kaiser Permanente of Georgia, Emory University School of Medicine, Atlanta, GA 30322, USA; 4 University of Miami Miller School of Medicine, Miami, FL 33136, USA; 5 Department of Medicine, University of California, Davis, Modesto, CA 95350, USA; 6 Geriatric Research Education and Clinical Center, VA Health Care System, Minneapolis, MN 55417, USA

**Keywords:** age, mortality, CKD, sarcopenia, skeletal muscle index

## Abstract

Low muscle mass (sarcopenia) is a prevalent and major concern in the aging population as well as in patients with chronic kidney disease (CKD). We hypothesized that sarcopenia is an independent predictor of incident and progressive CKD and increased mortality in older men and women (≥65 years) from the Cardiovascular Health Study. Sarcopenia was defined by bioimpedance-estimated skeletal muscle mass index (SMI) as a continuous variable and categorically (normal, class I, and class II). Cox regression hazard ratios (HRs) estimated the risk of incident and prevalent CKD and mortality in individuals with and without CKD. Low SMI was associated with increased prevalence of CKD in men (p<0.001), but lower prevalence of CKD in women (p=0.03). Low muscle mass was not associated with incident CKD or rapid CKD progression (>3 ml/minute/1.73m^2^/year decline in eGFR) in men, but was associated with lower risk of incident CKD in women ([adjusted RR=0.69, 95% (0.51,0.94)]. Low muscle mass (class II) was independently associated with higher mortality only in men [(adjusted HR=1.26, 95% (1.05,1.50)]. Neither definition of sarcopenia was associated with mortality in men or women with CKD. Further studies are needed to understand the mechanisms by which sarcopenia contributes to higher mortality in aging men.

## INTRODUCTION

Through regular physical activity, many anti-inflammatory cytokines produced and released into the circulation by skeletal muscle cells help maintain physiologic homeostasis [1]. For example, exercise has been shown to increase plasma levels of interleukin-6 (IL-6) and other anti-inflammatory cytokines such as interleukin-10 (IL-10). Loss of muscle cell volume, owing to the biological processes associated with aging, could create an inflammatory milieu. Such an environment could contribute to the development of distant organ dysfunction. Accumulating evidence also suggests that with older age and/or in diseased individuals, skeletal muscle may be involved in crosstalk with other organs [2–4], and therefore may play an important mechanistic role in kidney damage under catabolic conditions with age. Moreover, several lines of evidence have demonstrated a muscle-kidney crosstalk, due in part, by mitochondrial RNA secreted by the muscle via exosomes [5–7]. Thus, the catabolic influence on skeletal muscle with aging or disease results in maladaptive behavior of several factors associated with the acceleration of organ dysfunction.

Low muscle mass is common in patients with advanced chronic kidney disease (CKD) [8, 9]. Less is known about muscle mass in earlier stages of CKD and how muscle mass in early CKD impacts renal and cardiovascular outcomes longitudinally. This issue is of importance given the high prevalence of CKD in older (≥ 65years) adults [10, 11]. In the present study we examined the association of muscle mass with prevalent CKD in a cohort of older community-dwelling participants from the Cardiovascular Health Study (CHS). We also tested the hypothesis that low muscle mass is an independent predictor of: 1) incident CKD, 2) rapid CKD progression, or 3) mortality. Considering the effects of sex on muscle mass and CKD, we tested these hypotheses separately in men and women.

## RESULTS

### Population characteristics

**Figure 1:**
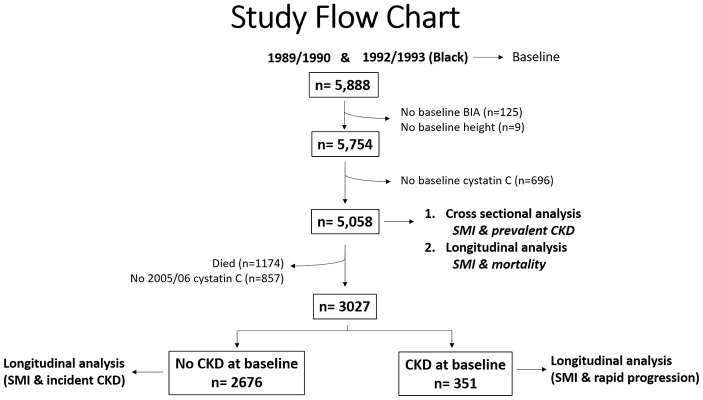
**Study Flow Chart.**

There were 5,888 individuals who had sufficient data from two CHS cohorts; year 2 (1989/90 cohort) and year 5 (1992/93 cohort; blacks only). Of the 5888 eligible individuals, 125 had missing bioelectrical impedance (BIA) values, and 9 had missing height measurements (Figure 1). Therefore, skeletal muscle mass index (SMI) was computed for 5754 participants (98%). Of these, 5,508 had cystatin-C estimated glomerular filtration rate (eGFR) values available and were included in the cross-sectional analysis and in the longitudinal mortality analysis. As shown in Figure 1, 3027 individuals were alive at the 2005/06 visit and had cystatin C values available. Of those, 2676 individuals did not have CKD at baseline and thus, were included in the longitudinal incident CKD analysis. Further, 351 individuals had CKD at baseline and were included in the rapid CKD progression analysis. Median follow-up time for incident CKD and CKD progression was 7 years.

### Baseline characteristics

Baseline characteristics are highlighted in Tables 1A and 1B for men and women and are shown according to SMI quartiles. Additionally, baseline characteristics between men and women can be viewed in Supplementary Table 1. In women, those with lower SMI were more likely to be older in age, less likely to be diabetic and black, and had lower fasting glucose and higher high-density lipoprotein (HDL)-cholesterol and eGFR values. Cardiovascular events, low density lipoprotein (LDL) and systolic blood pressure (SBP) did not differ across SMI quartiles in women. In men, those with a lower SMI were more likely to be older in age, have lower eGFR and fasting glucose values, and higher HDL-cholesterol values. Black race, cardiovascular events, hypertension, diastolic blood pressure (DBP), and SBP were not different across SMI quartiles in men.

**Table 1A:** Baseline characteristics in older men according to SMI quartile.

**SMI quartiles**					
**Characteristic**	**Q1 N=615**	**Q2 N=615**	**Q3 N=614**	**Q4 N=615**	**P value**
Age (years)	75±6	74±6	72±5	72±5	<0.001
Black Race (%)	15%	13%	10%	16%	0.03
Educational level (≥12 yrs)	45%	52%	48%	46%	0.04
Smoking					<0.001
% Current	16%	13%	9%	7%	
% Former	53%	56%	63%	55%	
% Never	32%	32%	29%	38%	
Alcohol (%)					<0.001
>7 drinks per week	19%	18%	19%	14%	
0 drinks per week	41%	40%	40%	46%	
1-7 drinks per week	40%	42%	42%	40%	
Diabetes Mellitus (%)	16%	16%	18%	26%	<0.001
Chronic kidney disease (%)	29%	21%	17%	19%	<0.001
Hypertension (%)	56%	52%	57%	59%	0.10
Coronary heart disease (%)	26%	25%	25%	26%	0.96
Stroke (%)	7%	4%	7%	5%	0.20
SBP (mmHg)	135±22	136±22	136±22	137±21	0.07
DBP (mmHg)	72±12	72±12	72±11	72±11	0.48
LDL- cholesterol (mg/dL)	124.6±34.7	122.3±31.3	125.4±33.3	121.2±32.9	0.25
HDL- cholesterol (mg/dL)	49.7±13.5	48.2±13.0	47.2±12.5	45.4±11.5	<0.001
Fasting glucose (mg/dL)	111±36	113±39	113±33	119±39	<0.001
Cystatin C (mg/dL)	1.17±0.46	1.08±0.28	1.05±0.26	1.08±0.32	<0.001
eGFR (mL/ min/1.73 m^2^)	70.5±20.1	75±18.3	76.5±17.5	75.1±18.4	<0.001
HOMA-IR	4.3±8.8	5.9±19.2	4.9±6.5	6.6±16.3	0.02

Data are shown as either mean +/- standard deviation or % for continuous and categorical variables, respectively. SMI: skeletal muscle mass index; Q: quartile; SBP: systolic blood pressure; DBP: diastolic blood pressure; LDL: low density lipoprotein; HDL: high density lipoprotein; eGFR: estimated glomerular filtration rate; HOMA-IR: Homeostatic Model Assessment of Insulin Resistance.

**Table 1B:** Baseline characteristics in older women according to SMI quartiles.

**Characteristic**	**Q1 N=824**	**Q2 N=824**	**Q3 N=823**	**Q4 N=824**	**P-val**
Age (years)	74±6	73±5	72±5	72±4.85	<0.001
Black Race (%)	12%	15%	17%	23%	<0.001
Educational level (≥ 12 years)	44%	42%	42%	34%	<0.001
Smoking					<0.001
% Current	16%	13%	9%	7%	
% Former	30%	33%	30%	30%	
% Never	55%	53%	58%	63%	
Alcohol (%)					<0.001
>7 drinks per week	10%	11%	8%	4%	
0 drinks per week	54%	53%	54%	64%	
1-7 drinks per week	36%	36%	38%	32%	
Diabetes Mellitus (%)	7%	10%	16%	25%	<0.001
Chronic kidney disease (%)	29%	21%	17%	19%	<0.001
Hypertension (%)	59%	56%	59%	69%	<0.001
Coronary heart disease (%)	15%	14%	16%	17%	0.50
Stroke (%)	3%	3.5%	3.3%	2.9%	0.90
SBP (mmHg)	137±24	136±22	136±21	139±21.7	0.14
DBP (mmHg)	69±11	70±11	69±11	71±11	0.02
LDL-cholesterol (mg/dL)	135.4±37.2	134.4±35.9	134.7±37.8	133.3±35.3	0.29
HDL-cholesterol (mg/dL)	63.0±16.77	60.8±16.2	57.9±15.5	54.4±13.8	<0.001
Fasting glucose (mg/dL)	101±27	105±30	111±38	120±48	<0.001
Cystatin C (mg/dL)	1.03±0.39	1±0.25	1.02±0.29	1.07±0.37	0.01
eGFR (mL/min/1.73 m^2^)	80.8±20.6	81.7±19.6	80.1±19.9	77.2±20.2	<0.001
HOMA-IR (units)	3.3±3.8	4.3±14.3	5.6±17.3	8.1±18.4	<0.001

Data are shown as either mean +/- standard deviation or % for continuous and categorical variables, respectively. SMI: skeletal muscle mass index; Q: quartile; SBP: systolic blood pressure; DBP: diastolic blood pressure; LDL: low density lipoprotein; HDL: high density lipoprotein; eGFR: estimated glomerular filtration rate; HOMA-IR: Homeostatic Model Assessment of Insulin Resistance

### Cross-sectional association between CKD and SMI quartiles at baseline

**Figure 2:**
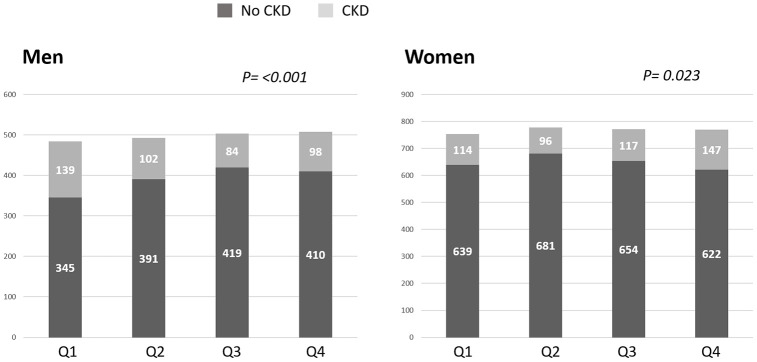
**Prevalence of chronic kidney disease (CKD) as according to skeletal muscle index (SMI) quartiles.** In men, the prevalence of CKD defined as cystatin C eGFR <60 mL/min/1.73 m^2^ increased with lower SMI such that the men with the lowest SMI quartile (Q1) had the highest prevalence of CKD. In women, the prevalence of CKD increased with higher SMI quartiles. Q: quartile.

As represented in Figure 2, in the unadjusted analysis, for each unit decrease in SMI there was a lower (RR=0.95 [95% C.I. 0.91,1.00]; p=0.04) prevalence of CKD in women. In contrast, each unit SMI decrease was associated with a higher (RR=1.13; [95% C.I. 1.05,1.22]; p<0.01) prevalence of CKD in men.

We then evaluated whether there was an independent association between SMI and prevalent CKD. In men, after full adjustment for potential covariates, SMI was not associated with CKD. There was however a significant interaction term by race among men (Table 2). There was no association between SMI and CKD in black men. In non-black men, however, there was a tendency of SMI to associate with CKD, but this did not achieve statistical significance in the M2 adjusted model (p=0.06). Among women, lower SMI was associated with a lower RR of CKD in unadjusted and adjusted analyses. When stratifying for race, lower SMI was associated with a lower RR of CKD in non-black women; however, no association was observed between SMI and CKD in black women (Table 2).

**Table 2:** Cross-sectional association between baseline inverse SMI and CKD.

	**Men**		**Women**	
	**RR (95% C.I.)**	**P value**	**RR (95% C.I.)**	**P value**
All subjects				
M0	1.13 (1.05,1.22)	0.002	0.95 (0.91,1.00)	0.04
M1	1.04 (0.97,1.11)	0.33	0.92 (0.89,0.95)	<0.001
M2	1.05 (0.98,1.12)	0.20	0.94 (0.89,0.99)	0.01
Non-blacks				
M0	1.17 (1.07,1.28)	<0.001	0.89 (0.81,0.97)	0.009
M1	1.07 (0.98,1.16)	0.13	0.81 (0.74,0.88)	<0.001
M2	1.09 (0.99,1.18)	0.06	0.87 (0.80,0.95)	0.003
Blacks				
M0	1.03 (0.91,1.17)	0.62	0.99 (0.90,1.09)	0.86
M1	0.97 (0.88,1.08)	0.57	0.98 (0.90,1.06)	0.55
M2	0.96 (0.86,1.10)	0.50	1.01 (0.88,1.15)	0.93

SMI: skeletal mass index, modeled as a continuous variable. CKD was defined as eGFR <60 mL/min/1.73m^2^.

M0: unadjusted

M1: adjusted for age and race

M2: adjusted for M1 + educational level, smoking status, alcohol use, h/o diabetes mellitus, h/o hypertension, h/o myocardial infarction, stroke, or congestive heart failure, systolic blood pressure, diastolic blood pressure, LDL-cholesterol, HDL- cholesterol, fasting glucose, and HOMA-IR.

### Muscle mass as a predictor of incident CKD and CKD progression

In men, there was no association between SMI and incident CKD in the unadjusted or adjusted analyses (adjusted RR for incident CKD was 1.09 [95% C.I. 0.94, 1.27; p=0.25]). In women, SMI was associated with a reduced incidence of CKD (M0: RR for incident CKD. WAS 0.84 [95% C.I. 0.73, 0.92]; p<0.02) and (M1; RR was 0.82 [95% C.I. 0.74, 0.90]; p<0.001) respectively. However, after adjusting for the variables in the M2 analysis there was no association between baseline SMI and incident CKD in women (p=0.09). Similar findings were noted when SMI was modeled as a categorical variable according to sarcopenia class/category (Table 3).

**Table 3:** Association between sarcopenia class and incident CKD.

	**Men**		**Women**	
	**RR (95% C.I.)**	**P value**	**RR (95% C.I.)**	**P value**
**Class I**	1.13 (0.69,1.84)	0.64	0.75 (0.51,1.12)	0.16
**Class II**	1.27 (0.67,2.41)	0.47	0.73 (0.34,1.57)	0.42
**M1-Class I**	1.13 (0.75,1.71)	0.56	0.69 (0.51,0.94)	0.02
**M1-Class II**	1.11 (0.64,1.91)	0.71	0.56 (0.31,1.02)	0.06
**M2-Class I**	1.29 (0.84,1.97)	0.24	0.83 (0.61,1.13)	0.23
**M2-Class II**	1.35 (0.77,2.37)	0.29	0.73 (0.41,1.29)	0.28

Class I sarcopenia was defined as SMI of 8.51–10.75 kg/m^2^ for men and 5.76–6.75 kg/m^2^ for women. Class II sarcopenia was defined as SMI ≤ 8.50 kg/m^2^ for men and ≤ 5.75 kg/m^2^ for women.

M0: unadjusted

M1: adjusted for age and race

M2: adjusted for M1 + educational level, smoking status, alcohol use, h/o diabetes mellitus, h/o hypertension, h/o myocardial infarction, stroke, or congestive heart failure, systolic blood pressure, diastolic blood pressure, LDL-cholesterol, HDL- cholesterol, fasting glucose, and HOMA-IR

Of the 351 participants with CKD at baseline, 46 had rapid decline in kidney function at the year 2005/06 follow-up visit (defined as eGFR decline >3 mL/min/1.73m^2^ per year). In men, lower SMI was not associated with rapid decline in kidney function in the unadjusted analysis (RR was 0.88; 95% C.I.0.64, 1.20; p=0.41) or in the multivariate adjusted model. Similar findings were observed in women (RR for rapid decline in kidney function was 1.11; 95% C.I. 0.74, 1.66; p=0.62 in unadjusted analysis and remained non-significant in the multivariate models).

### Association between sarcopenia and mortality in older men and women with and without CKD

**Figure 3:**
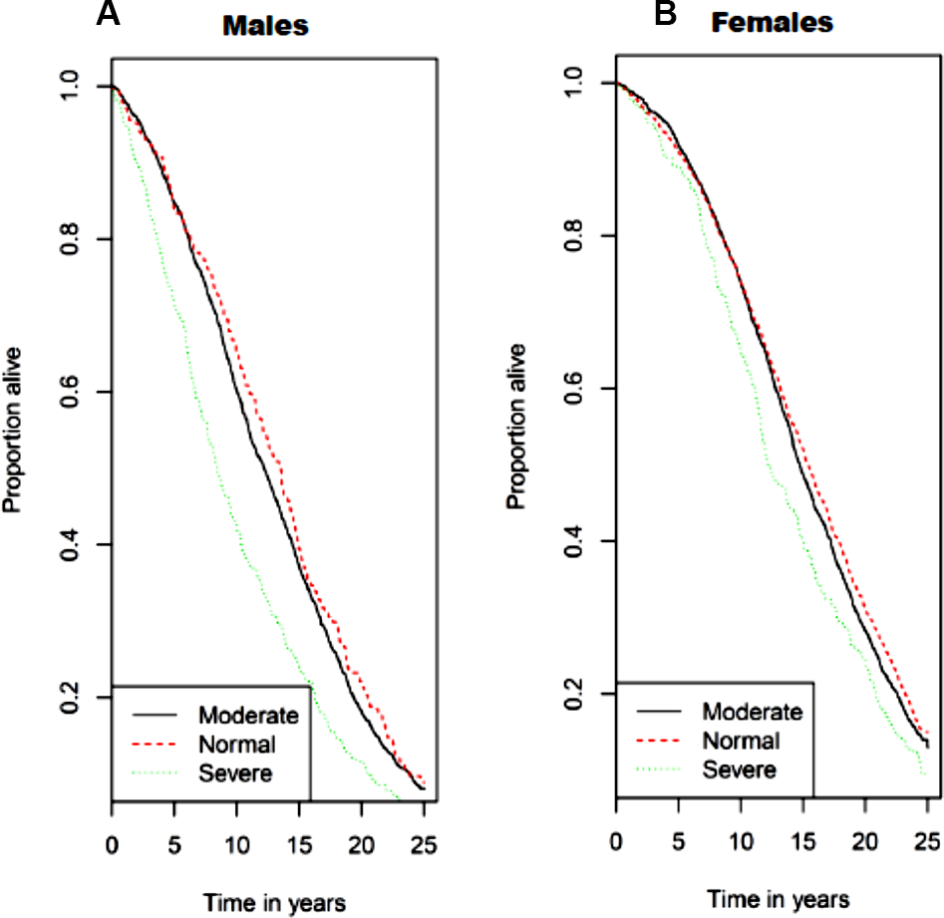
****Kaplan−Meier survival curves for all-cause mortality by sarcopenia class/category with (**A**) for men and (**B**) for women. Normal skeletal muscle mass was defined as SMI ≥ 10.76 kg/m^2^ for men and ≥ 6.76 kg/m^2^ for women. Class I sarcopenia was defined as SMI 8.51-10.75 kg/m^2^ for men and 5.76-6.75 kg/m2 for women. Class II sarcopenia was defined as SMI ≤ 8.50 kg/m^2^ and ≤ 5.75 kg/m^2^ for men and women, respectively.

In unadjusted analysis, men with class II sarcopenia exhibited greater mortality rates compared to class I sarcopenia and normal SMI values (Figure 3). Cox regression analysis (shown in Table 4A) demonstrated that men with class II sarcopenia had higher mortality rates in both multivariate models. In contrast, while women with class II sarcopenia had higher mortality rates than those with SMI in the normal range in the unadjusted analysis, no association was observed between class I and class II sarcopenia and mortality in either multivariate models (Table 4A). Of note, there was no interaction of CKD status at baseline regarding the association of sarcopenia and mortality in men or women. Furthermore, in the subgroup of older adults with CKD at baseline (n=351) there was no association of mortality and lower SMI (as a continuous or categorical variable), as shown in Table 4B.

**Table 4A:** Association between sarcopenia class and mortality.

	**Men**		**Women**	
	**HR (95% C.I.)**	**P value**	**HR (95% C.I.)**	**P value**
Class I	1.09 (0.95,1.25)	0.24	1.06 (0.98,1.15)	0.15
Class II	1.59 (1.35,1.89)	<0.001	1.30 (1.14,1.49)	<0.001
M1-Class I	1.02 (0.89,1.17)	0.81	0.97 (0.89,1.05)	0.42
M1-Class II	1.24 (1.05,1.47)	0.01	0.94 (0.82,1.08)	0.39
M2-Class I	1.06 (0.91,1.22)	0.47	1.02 (0.94,1.11)	0.64
M2-Class II	1.26 (1.05,1.50)	0.01	1.04 (0.90,1.21)	0.60

Class I sarcopenia was defined as SMI of 8.51–10.75 kg/m^2^ for men and 5.76–6.75 kg/m^2^ for women. Class II sarcopenia was defined as SMI ≤ 8.50 kg/m^2^ for men and ≤ 5.75 kg/m^2^ for women.

M0: unadjusted

M1: adjusted for age and race

M2: adjusted for M1 + educational level, smoking status, alcohol use, h/o diabetes mellitus, h/o hypertension, h/o myocardial infarction, stroke, or congestive heart failure, systolic blood pressure, diastolic blood pressure, LDL-cholesterol, HDL- cholesterol, fasting glucose, and HOMA-IR

**Table 4B:** Association between sarcopenia class and mortality in adults with CKD.

	**Men**		**Women**	
	**HR (95% C.I.)**	**P value**	**HR (95% C.I.)**	**P value**
Class I	1.11 (0.83,1.50)	0.48	0.98 (0.80,0.19)	0.80
Class II	1.29 (0.92,1.81)	0.14	1.35 (0.98,1.85)	0.06
M1-Class I	1.10 (0.82,1.49)	0.52	0.96 (0.79,1.18)	0.71
M1-Class II	1.15 (0.82,1.62)	0.42	0.95 (0.69,1.32)	0.76
M2-Class I	1.13 (0.82,1.57)	0.46	0.92 (0.74,1.15)	0.47
M2-Class II	1.20 (0.82,1.74)	0.35	0.98 (0.69,1.38)	0.88

Class I sarcopenia was defined as SMI of 8.51–10.75 kg/m^2^ for men and 5.76–6.75 kg/m^2^ for women. Class II sarcopenia was defined as SMI ≤ 8.50 kg/m^2^ for men and ≤ 5.75 kg/m^2^ for women.

M0: unadjusted

M1: adjusted for age and race

M2: adjusted for M1 + educational level, smoking status, alcohol use, h/o diabetes mellitus, h/o hypertension, h/o myocardial infarction, stroke, or congestive heart failure, systolic blood pressure, diastolic blood pressure, LDL-cholesterol, HDL- cholesterol, fasting glucose, and HOMA-IR

## DISCUSSION

In this study several important findings were observed. First, low muscle mass was associated with an increased prevalence of CKD in older community dwelling men. Conversely, low muscle mass was associated with a lower prevalence of CKD in older community dwelling women. Second, contrary to our hypothesis, low muscle mass was not predictive of incident CKD or CKD progression in older men or women. However, low muscle mass was independently associated with increased risk of mortality in older men. Although severely reduced muscle mass was associated with increased mortality in older women in the unadjusted analysis, this was not significant in the multivariate models. Lastly, we found no association between muscle mass and mortality in men or women with CKD.

Previous studies have identified over 3,000 genes that are differentially expressed in men and women, which are linked to muscle mass loss [12]. Age-related decline in skeletal muscle mass and function is also confounded by disease status, such as CKD [8]. Patients with CKD exhibit a catabolic state, which is known to be associated with protein wasting and with multiple metabolic imbalances due, in part, to elevated uremic toxins [13, 14]. Data from the Framingham Heart Study suggest that the longitudinal decline in lean muscle mass with age is a consequence of different underlying mechanisms in men and women such as withdrawal of anabolic stimuli in men and an increase in catabolic stimuli (such as IL-6) in women [15]. Other data suggest that sarcopenia is mediated by the catabolic influence of myostatin in men, whereas, in women sarcopenia may be mediated by reduced insulin-like growth factor 1 [15, 16]. Hence, it is plausible that catabolic environments, such as that associated with CKD, would affect skeletal muscle mass differently in men and women. This may explain why low muscle mass was associated with a higher prevalence of CKD in men but with a lower prevalence of CKD in women in the present analysis.

An analysis from the National Health and Nutrition Examination Survey (NHANES) reported that sarcopenia is more prevalent among the general population of men and women who have reduced GFR [17]. Our present analysis is in partial agreement with this evidence, as older men with low muscle mass had a higher prevalence of CKD. Interestingly, our data are in agreement with data from the Korean NHANES in which sarcopenia (measured via dual x-ray absorptiometry; DXA) was prevalent in both men and women (P<0.001) with CKD, but the stage of CKD was only associated with increased prevalence of sarcopenia in men and not women [18]. Similar findings were observed in a small study of men and women with CKD, where more men with CKD had low muscle mass compared to women with CKD [19]. Collectively, these findings and ours suggest that sex specific differences exist in relation to loss of muscle mass and decreased eGFR.

The sex hormones estrogen and testosterone have been implicated in skeletal muscle growth, fiber size, and contractile function [20]. Some reports highlight enhanced contractile function and increased β-oxidative gene expression in men supplemented with estrogen and enhanced muscle growth in women treated with testosterone. Hence, men and women may respond differently to catabolic conditions because of their hormonal profiles [20]. Along these lines, testosterone is a potent anabolic factor promoting muscle protein synthesis and muscular regeneration and is found in much higher concentrations in young men compared to young women. However, progressive aging and/or the combination of aging with CKD results in greater reductions in testosterone in men compared to women. Consequently, this may result in more rapid reductions in muscle mass in men rendering them more sensitive to the effects of CKD. Indeed, in the present analysis, men were of older age, and there was nearly a two-fold higher percentage of older men who exhibited severe sarcopenia relative to their age-matched female counterparts.

In this analysis, we found a significant interaction of race on the association of muscle mass and CKD [21]. Several epidemiological studies demonstrate large variability in the prevalence of sarcopenia between racial/ethnic subpopulations of older adults. with non-Hispanic black men and women exhibiting the lowest prevalence of sarcopenia [22, 23]. This is consistent with our findings that muscle mass is preserved in older black men even in the presence of CKD. It has been shown that black men have modest but significantly higher free testosterone levels compared with white men [24]. Black men are also known to exhibit higher estradiol and estrogen levels than white men [25]. Importantly, the weight of evidence from human and animal studies demonstrates that estrogen and estradiol hormone replacement therapy has significant beneficial effects in maintaining muscle mass and function, and may help offset age-related loss of muscle mass [26]. Hence, our findings may be explained by the differences in sex hormone levels across racial groups.

Another important aim of the present study was to test the hypotheses that, among older men and women, low muscle mass is an independent predictor for developing CKD (incident CKD) and, among those with CKD, that low muscle mass independently predicts rapid kidney disease progression. Our findings did not support these hypotheses, as it was shown that muscle mass did not predict incident CKD or CKD progression in men and women. It is highly likely that these findings are in part due to competing risk of death in this population of older adults. In the present analysis, we found that older men with class II sarcopenia had lower survival rates than men with the other categories of sarcopenia after adjusting for age, race and cardiovascular risk factors. Interestingly, no such associations were evident in older women after adjusting for race and cardiovascular disease (CVD) risk factors. One potential reason behind these findings is that men have been demonstrated to be more vulnerable to muscular oxidative damage than women, and this has been attributed to differences in fiber type composition [27, 28]. Moreover, increased levels of oxidative stress produced by reactive oxygen species has been shown to be associated with skeletal muscle mass loss in predominantly type II fibers found in higher concentration in men versus women, and this may be due in part, to reduced vascular function to adequately supply/replenish oxygen and essential nutrients for muscle tissue growth and repair.

Of note, we found no association between muscle mass and mortality in individuals with CKD in this analysis. While these findings are limited by the small number of individuals with CKD at baseline, our findings are consistent with the findings of Androga et al. in NHANES 1999-2004, which showed that sarcopenia was associated with increased mortality in individuals without CKD but not in those with CKD [29]. Interestingly, the data from NHANES indicated that sarcopenia was associated with mortality only in individuals with CKD who were obese. The mean BMI was 26.4 and 26.9 kg/m^2^ in men and women of the CHS, respectively (consistent with overweight but not obese). This may explain the lack of an association between sarcopenia and mortality in CKD. Our findings are in contrast to the report by Pereira et al. which showed that sarcopenia, defined as reduced handgrip strength and low SMI, was an independent predictor of mortality in non-dialysis-dependent patients with stages 3-5 CKD [30]. It is possible that our findings differed due to the lower prevalence of advanced CKD (stage 4 or 5) in the CHS.

Our investigation is not without limitations. First, analyses were conducted in a survival cohort, and as such, few participants had rapidly advancing CKD. In addition, the small number of participants with baseline CKD likely impaired our ability to evaluate the impact of sarcopenia on CKD progression or death in this group of patients. Second, although sarcopenia is defined by both loss of muscle mass and muscle function, our study investigated one operational definition of sarcopenia, which was the quantification of muscle mass using the method of BIA. Sarcopenia is defined as having both loss of muscle mass and muscle function. Here, sarcopenia was defined as low muscle mass. However, prior studies have investigated the association of sarcopenia, defined by impaired muscle function (but not muscle mass), with incident CVD and mortality in individuals with CKD. Roshanravan et al. [31] showed that impaired physical performance (one operational definition of sarcopenia) of the lower extremities is common in CKD and strongly associates with all-cause mortality in this population. However, Roshanravan et al. did not explore whether sex differences modified this association and as such, the underlying mechanisms behind this association are unclear. In addition, our analyses were based on a single baseline measure of muscle mass and did not account for changes in muscle mass over time, including those that may be secondary to changes in diet or nutrition. Future works should evaluate whether such lifestyle changes result in significant change in skeletal muscle mass or if such changes over time (for example increased skeletal muscle mass) would improve long term outcomes, such as mortality. Lastly, muscle mass was measured using BIA. Though BIA may not be as accurate or reliable as other methods to evaluate body composition such as DXA, computed axial tomography, air displacement plethysmography (i.e., Bod-POD) and magnetic resonance imaging, BIA, DXA and Bod-POD are highly correlated, even when body composition was actively changing because of a severe diet [32]. In addition, BIA is a simple-to-use, affordable and reliable device, and is one of the most commonly used methods to evaluate body composition in clinical settings.

In conclusion, we report that low muscle mass is associated with increased prevalence of CKD in older community dwelling men, whereas this was inversely associated in women. While low muscle mass was not predictive of incident CKD or CKD progression in older men or women, low muscle mass was independently associated with increased risk of mortality in older men. Further research is needed to confirm our findings and to better understand the mechanisms that underlie the association between low muscle mass and prevalent CKD and mortality.

## MATERIALS AND METHODS

### Study population

5888 community dwelling men and women aged 65 years or older enrolled in the 2-phase prospective cohort CHS (1989-1993). These included an initial cohort of 5201 enrolled in 1989/90 and an additional 687 African-American participants enrolled in 1992/93. CHS participants were randomly sampled from Medicare eligibility lists at four U.S. sites: University of California, Davis (Sacramento County, Sacramento, CA), Johns Hopkins University (Washington County, Hagerstown, MD), Wake Forest University School of Medicine (Forsyth County, Winston-Salem, NC), and University of Pittsburgh (Allegheny County, PA) [27]. The aims of CHS were to determine the importance of conventional cardiovascular disease (CVD) risk factors and to identify new CVD risk factors, especially those that may be protective and modifiable in a large cohort of community dwelling older adults. Informed consent was obtained from all participants and approved by the institutional review boards of the 4 clinical sites. Full details of the design, recruitment and procedures of the CHS are available in previously published work [33].

### Assessment of body composition and skeletal muscle index

Body weight and height were measured at participant baseline to the nearest 0.1 kg and 0.1 cm using standardized equipment and procedures. Body mass index was calculated as weight/height^2^ (kg/m^2^). BIA resistance (Ohms) was obtained using a TVI-10 Body Composition Analyzer (Danninger Medical Technology, Inc., Columbus, OH) with an operating frequency of 50 kHz at 800 uA. Whole-body BIA measurements were taken between the right wrist and ankle with the subject in a supine position after an overnight fast [34]. Muscle mass in kg was calculated based on BIA utilizing a calculation that was developed and cross-validated against magnetic resonance imaging measures of whole-body muscle mass in men and women varying in age and adiposity [35]. This calculation has been used successfully in previous epidemiological studies of sarcopenia and disability [34, 36, 37].

Skeletal muscle mass index (SMI) was calculated using the BIA equation developed by Janssen and colleagues [35]: skeletal muscle mass (kg) = ((height^2^/BIA - resistance x 0.401) + (sex x 3.825) + (age x -0.071)) + 5.102, where height is in cm, BIA-resistance is in ohms, sex is 1 for men and 0 for women, and age is in years. Absolute skeletal muscle mass (kg) was converted to percentage skeletal muscle mass (muscle mass/body mass X 100) and termed SMI. SMI was used because it adjusts for stature and the mass of non-skeletal muscle tissue (fat, organ, bone). Most mobility tasks and activities of daily living are influenced by body mass.

### Sarcopenia classification

The presence of sarcopenia was based on the following disability-related SMI thresholds [36]:

1. Normal skeletal muscle mass: men ≥ 10.76 kg/m^2^, women ≥ 6.76 kg/m^2^;
2. Class I sarcopenia: men 8.51–10.75 kg/m^2^, women 5.76–6.75 kg/m^2^; or
3. Class II sarcopenia: men ≤ 8.50 kg/m^2^, women ≤ 5.75 kg/m^2^ [37]

### Outcome measures

Incident CKD was defined as cystatin C eGFR <60 mL/min per 1.73 m^2^ during visit 2005/06 [38]. eGFR was based on the CKD Epidemiology Collaboration formula and was calculated using the equation: eGFR = 76.7 x cystatin C [mg/L]^−1 19^ [39]. Cystatin C has been suggested to be better than creatinine as a marker for kidney function when evaluating issues related to muscle mass because creatinine is a byproduct of muscle metabolism [40]. In this study, cystatin C was measured at baseline (1989/90 and 1992/93) and (2005/06). CKD progression was defined as annualized loss of eGFR >3 mL/min/1.73 m^2^ [41] from baseline to 2005/06, as based on previous data from the CHS [42]. Death from all causes was adjudicated by the CHS Events Subcommittee as previously detailed [43]. Follow-up for mortality continued until death, loss to follow-up, or until 6/30/2014.

### Analyses

We compared sex-specific baseline characteristics across SMI quartiles using trend tests for continuous variables and Chi-squared tests for categorical variables. First, we examined the cross-sectional association between prevalent CKD and SMI quartiles utilizing Poisson regression models. Next, we evaluated the potential association between baseline SMI and incident CKD. To evaluate the association between low muscle mass and the designated outcomes, inverse SMI was modeled as a continuous variable and as sarcopenia categories. Of note, this analysis was limited to the 3336 participants who had eGFR >60 mL/ min/1.73 m^2^ at baseline and were alive with available cystatin C levels at the 2005/06 visit. We utilized Poisson regression modeling with a time offset to accommodate the different time interval between baseline and year 9 for the initial CHS cohort (enrolled 1989/90) and the African-American cohort (enrolled 1992/93). For those individuals with evidence of CKD at baseline, we evaluated the potential association between SMI and rapid kidney function decline. Considering the competing risk of mortality with incident CKD and CKD progression [44], we examined the association of baseline SMI with mortality using Cox hazard models to estimate hazard ratios (HR). We subsequently evaluated the interaction term with CKD status (defined as eGFR <60 mL/min/1.73 m^2^) and conducted stratified analysis according to CKD status in the men and women. The study flow chart for this analysis is shown in Figure 1.

For all analyses, we used two nested models: M1 was adjusted for age and race. M2 was additionally adjusted for educational level, current alcohol use, history of diabetes, history of hypertension, baseline CVD (defined as self-reported myocardial infarction, stroke, or congestive heart failure), SBP, DBP, LDL-cholesterol, HDL-cholesterol, fasting glucose, and HOMA-IR: Homeostatic Model Assessment of Insulin Resistance (HOMA-IR). We used splines in generalized additive models to address the functional form of continuous inverse SMI in the models; we found no meaningful departures from linearity. P values were not adjusted for multiple testing.

## Supplementary Material

Supplementary Table 1
