# Supplementary material for: Association of skeletal muscle mass, kidney disease and mortality in older men and women: the cardiovascular health study
Source: Aging (Albany NY). 2020 Nov 2;12(21):21023–36. doi: 10.18632/aging.202135 (PMC7695366; doi:10.18632/aging.202135)
Supplement: Supplementary Table 1 [file aging-12-202135-s001..pdf]

SUPPLEMENTARY TABLE

Supplemental Table 1. Baseline characteristics in older men and women.

Characteristics	Total (n=5754)	Men (n=2459)	Women (n=3295)	P Value
Age	73±6	73±6	73±6	<0.01
Black Race (%)	15%	14%	17%	<0.01
Educational level (≥12 yrs)	44%	48%	41%	<0.01
Smoking				
% Current	12%	11%	12%	<0.01
% Former	42%	57%	31%	
% Never	47%	32%	57%	
Alcohol (%)				
>7 drinks per week	12%	17%	8%	<0.01
0 drinks per week	50%	42%	56%	
1-7 drinks per week	38%	41%	36%	
Diabetes Mellitus (%)	16%	19%	14%	<0.01
Chronic kidney disease (%)	18%	21%	15%	<0.01
Hypertension (%)	59%	56%	61%	<0.01
Coronary heart disease (%)	20%	25%	16%	<0.01
Stroke (%)	4%	6%	3%	<0.01
SBP (mmHg)	137±22	136±21	137±22	0.02
DBP (mmHg)	71±11	72±12	70±11	<0.01
LDL- cholesterol (mg/dL)	130±36	123±33	135 ±37	<0.01
HDL- cholesterol (mg/dL)	54±16	48±13	59±16	<0.01
Fasting glucose (mg/dL)	111±37	114±37	109±37	<0.01
Cystatin C (mg/dL)	1.1±0.34	1.1±0.34	1.0±0.33	<0.01
eGFR (mL/ min/1.73 m^2)	77.7±19.8	74.3±18.7	79.9±20.1	<0.01
HOMA-IR	5.4±14.3	5.4±13.8	5.4±14.7	0.84
SMI (kg/m^2)	8.04±1.83	9.56 ±1.34	6.91±1.21	<0.01

Data are shown as either mean +/- standard deviation or % for continuous and categorical variables, respectively. SBP: systolic blood pressure; DBP: diastolic blood pressure; LDL: low density lipoprotein; HDL: high density lipoprotein; eGFR: estimated glomerular filtration rate; HOMA-IR: Homeostatic Model Assessment of Insulin Resistance; SMI: Skeletal muscle mass index.
